# Retraction: The effect of copy number on α-synuclein’s toxicity and its protective role in Bax-induced apoptosis, in yeast

**DOI:** 10.1042/BSR20201912_RET

**Published:** 2025-08-13

This article is being retracted from *Bioscience Reports* at the request of the authors and the Editorial Board. This follows the receipt of a notification from a reader, alerting the Editorial Board to similarities between some of the data represented in the figures of this paper with those of other papers published by the same author group. This includes the Figure 9E β-actin panel and the Figure 3G c-Myc Ab panel from Akintade et al, 2020 (DOI: 10.1007/s11033-020-05736-5), the Figure 2I Actin panel and the Figure 8I Bax panel (after a vertical flip transformation) within this paper, and Figure 3G and Figure 9-IV-A from Derf et al, 2018 (DOI: 10.1021/acsomega.8b01154).

The Editorial Office identified further image similarities between figures within the manuscript. Specifically:

- Between Figure 1A SG Agar Plate, 4G SD Agar plate/1C, and 6A SD Agar Plate,
- Between Figure 2H (HA/3C) and 4D 1C
- Between Figure 3A SD Agar Plate (1^st^ and 2^nd^ row) and 8A SD Agar Plate (2^nd^ row), and between 3A SG Agar Plate (1^st^ row) and 8A SG Agar Plate (1^st^ row)
- Between 3C - 2c and 3 c panels
- between 4G SG Agar Plate/2C and 5F SG Agar Plate/2C
- Between grey colonies of Figure 3I 2C and 3C panels with Figure 9C Bax 1C and 2C panels, and between Figure 3I 1C and Figure 9C Bax 3C
- Between Figure 4A SD Agar Plate/1C and Figure 8A SD Agar Plate/B3C
- Between Figure 7H Bax western blot bands (darker background) and Figure 9E B-actin bands (after rotation) and 9J B-actin bands.
- Between Figure 9A SG Agar Plate Bax 1-copy and 9A SG Agar Plate Bax 3-copy (after rotation)
- Between Figure 10A SD Agar + Methionine and 10A SG Agar + 0.1 glucose (different image quality).

The authors responded to our concerns with corrected versions of all affected Figures as well as new western blot raw data. However, their explanations for the similarities did not sufficiently resolve our concerns, particularly with regards to the differences in image quality and background of similar images. Therefore, the journal no longer has confidence in the reliability or validity of the results presented in this paper. The authors wish to retract the article, and the Editorial Board agree with this decision.

